# Extraosseus Ewing's Sarcoma of the Forearm

**DOI:** 10.7759/cureus.9051

**Published:** 2020-07-07

**Authors:** Kuldeep Bansal, Avijeet Prasad, Pratyush Shahi, Apoorv Sehgal, Sushil Kamal

**Affiliations:** 1 Orthopaedics, University College of Medical Sciences and Guru Teg Bahadur Hospital, Delhi, IND

**Keywords:** ewing's sarcoma, extraosseous, forearm, wide local excision, neoadjuvant chemotherapy, radiotherapy, recurrence, amputation

## Abstract

A 22-year-old female presented with painful, progressive swelling of the right forearm for six months. Physical examination revealed a 7 cm x 5 cm firm, tender soft-tissue swelling over the anterior aspect of the right proximal forearm with normal overlying temperature. X-rays showed increased soft tissue shadow but without any bony involvement. Serum alkaline phosphatase, serum lactate dehydrogenase, and leukocyte count were raised. MRI of the right forearm revealed enhancing soft tissue lesion with internal hemorrhagic and necrotic components involving the flexor carpi radialis muscle. Core biopsy confirmed the diagnosis of extraosseus Ewing’s sarcoma. Neoadjuvant chemotherapy, wide local tumor excision, and adjuvant chemotherapy and radiotherapy were done. The patient then lost to follow-up and presented again after six months with a fungating mass and neurovascular involvement for which an above-elbow amputation was done. We, through this case report, aim to discuss the clinical and radiological findings, line of management, and the importance of early detection and treatment and a regular follow-up for extraosseus Ewing's sarcoma of the extremity.

## Introduction

Ewing’s sarcoma (ES) is a malignant round cell tumor without cellular differentiation. It is the second most common malignant bone tumor in children and young adults. However, it can be extraskeletal in origin in rare cases. Extraosseus Ewing's sarcoma (EES) commonly involves the paravertebral spaces, lower extremities, head and neck, and pelvis [1]. Upper extremity accounts for only 3% of all cases of extraskeletal ES [2].

We report a 22-year-old female with EES of the right forearm with recurrence and discuss the clinical and radiological findings, line of management, and the importance of early detection and treatment and regular follow-up.

## Case presentation

**Figure 1:**
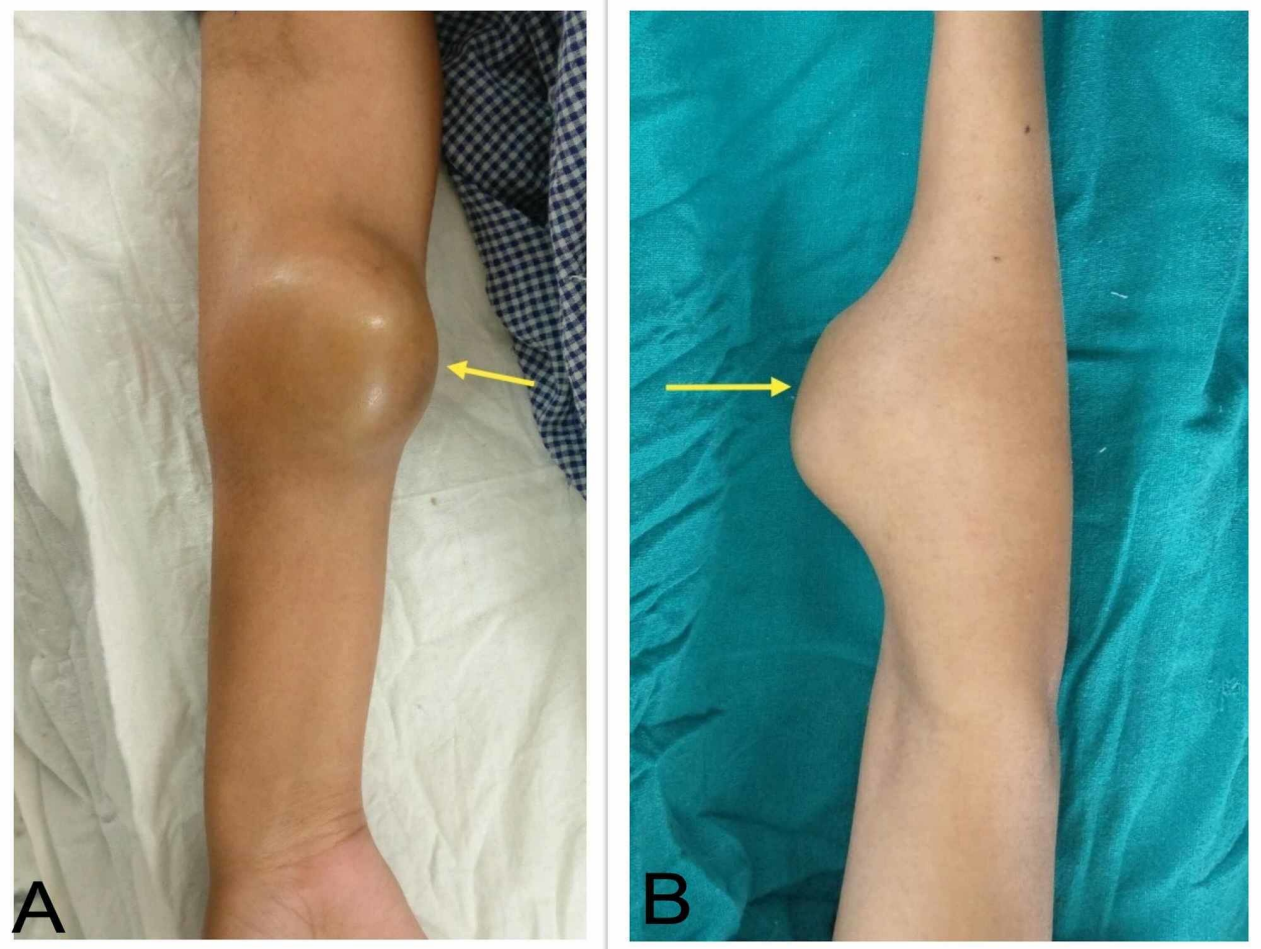
Clinical picture of the swelling. (A) From the front. (B) From the side.

A 22-year-old female presented with progressive swelling and pain in the right forearm for six months. It was not associated with trauma, fever, weight loss, or loss of appetite. Physical examination revealed a 7 cm x 5 cm firm swelling over the anterior aspect of the proximal third of the right forearm starting about 5 cm below the elbow joint line (Figure 1). The overlying temperature was normal, there was slight tenderness, and the surface was smooth and regular. The finger could be inserted between the swelling and underlying bone. Cardiorespiratory, abdominal, and neural examinations were unremarkable.

**Figure 2:**
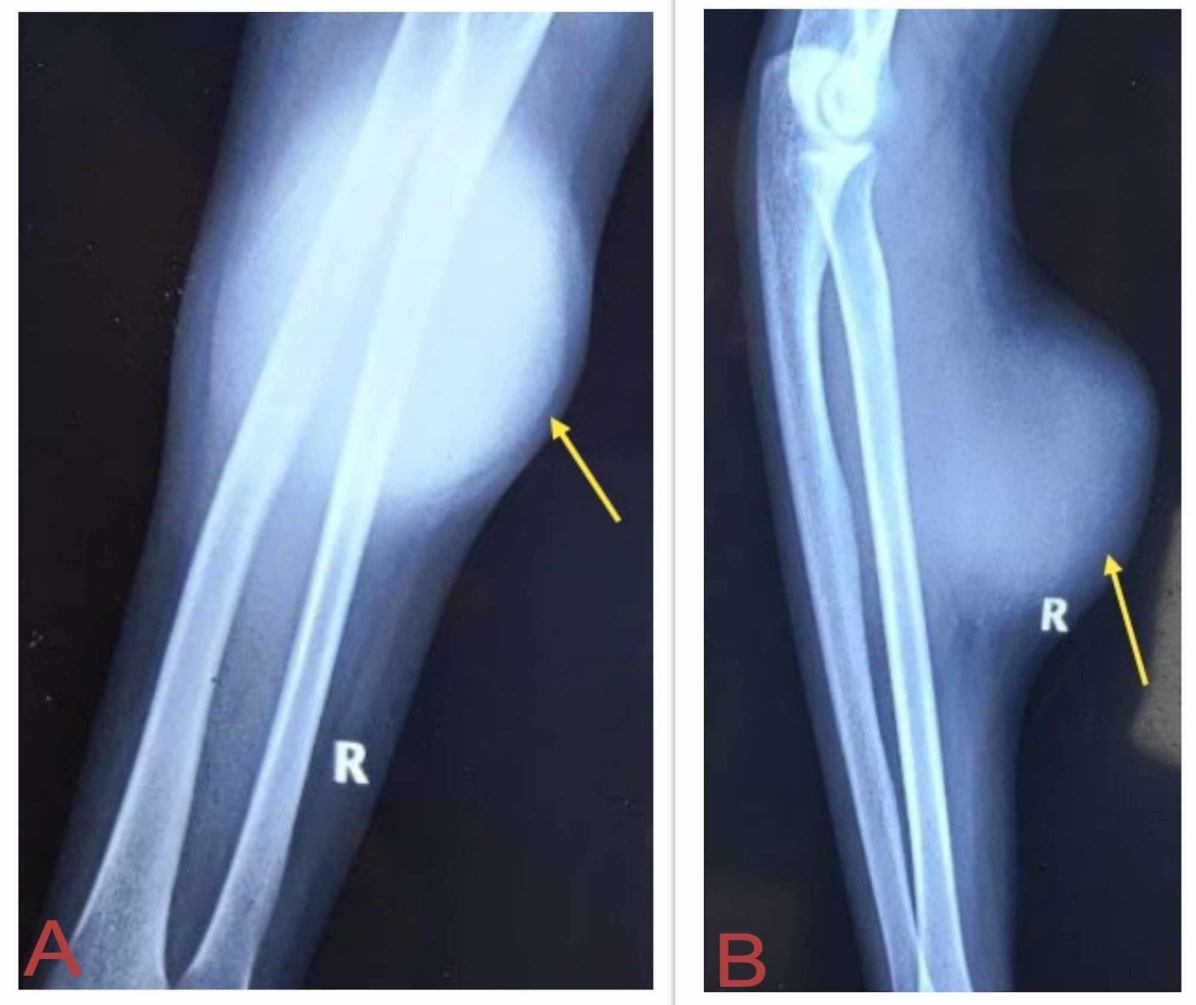
Preoperative X-rays of the right forearm showed increased soft tissue shadows with no bony involvement. (A) Anteroposterior view. (B) Lateral view.

X-rays of the right forearm showed increased soft tissue shadow without any bony involvement (Figure 2). The chest x-ray was normal. Serum alkaline phosphatase, serum lactate dehydrogenase, and leukocyte count were raised (454 IU/L, 836 U/L, and 11,200/mm^3^, respectively). Skeletal survey and contrast-enhanced computerized tomography (CECT) of the abdomen and pelvis were normal.

**Figure 3:**
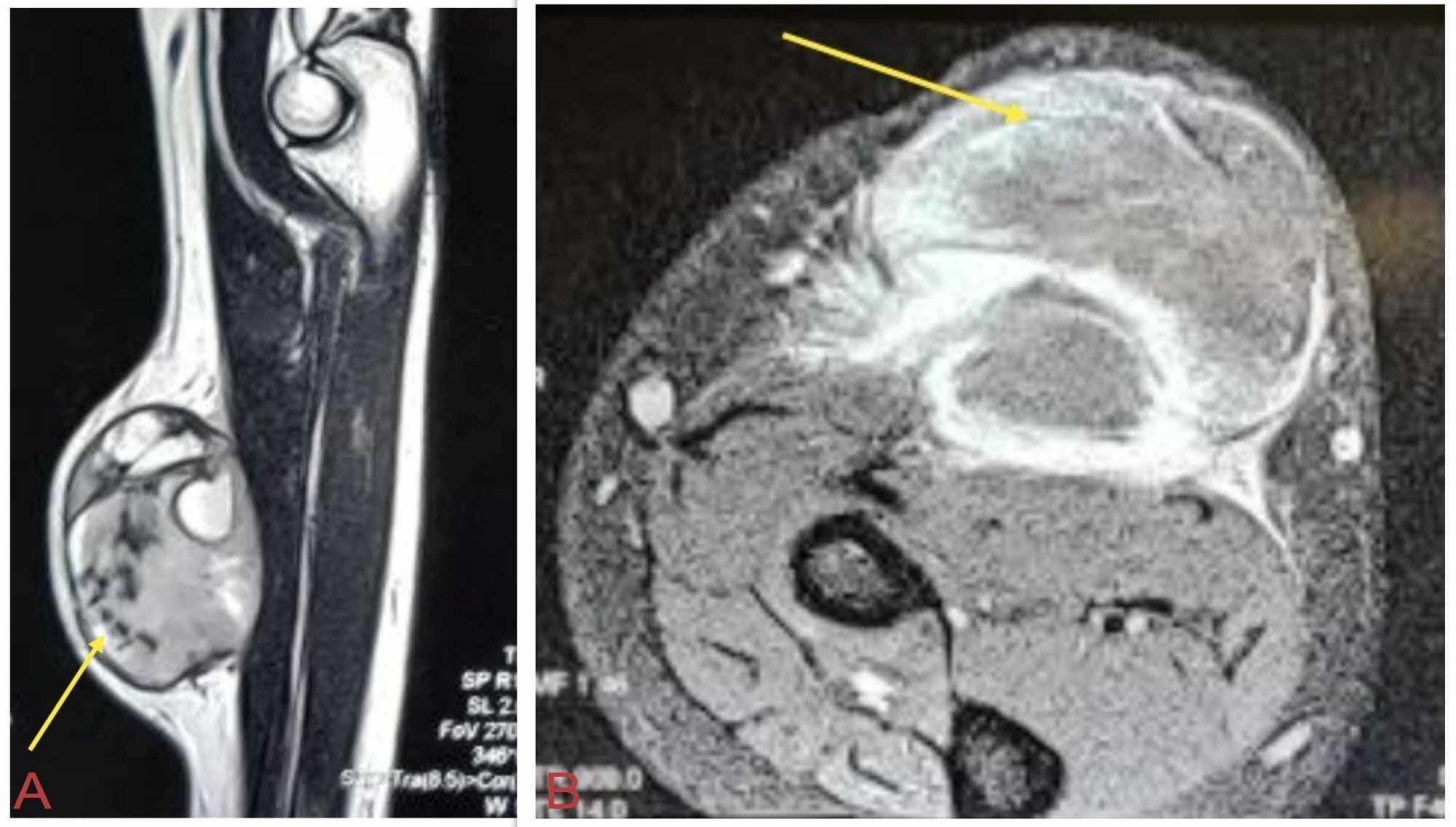
MRI shows subcutaneous enhancing soft tissue lesion with internal hemorrhagic and necrotic components involving the flexor carpi radialis muscle. (A) Sagittal section. (B) Transverse section.

MRI of the right forearm revealed enhancing soft tissue lesion with internal hemorrhagic and necrotic components involving the flexor carpi radialis muscle (Figure 3). Core needle biopsy showed round to oval monomorphic cells with high nuclear-cytoplasmic ratio arranged in pseudorosettes with CD99 positivity which suggested a small round cell tumor (EES). 

**Figure 4:**
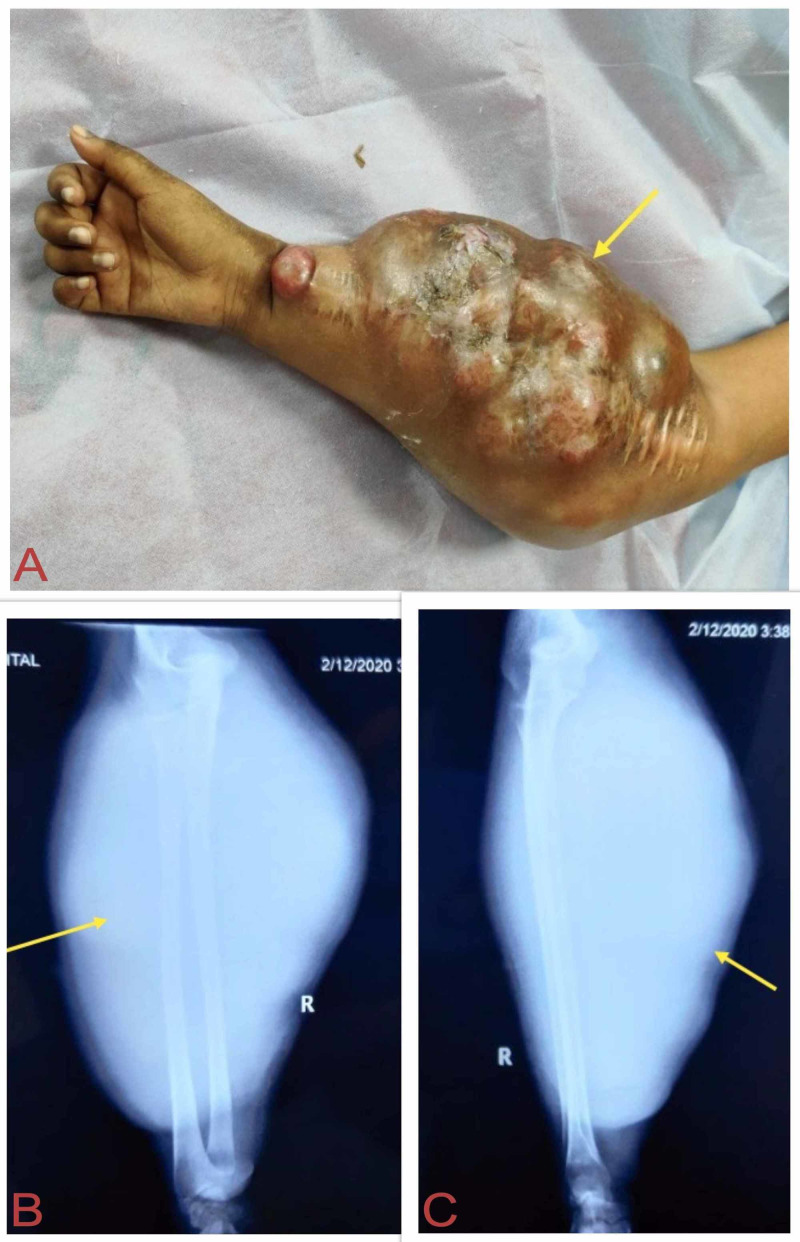
Recurrence after six months. (A) Fungating mass involving the whole of right forearm. (B, C) Anteroposterior and lateral view X-rays show massively increased soft tissue shadows.

The case was discussed in the multidisciplinary tumor board of the institution, and neoadjuvant VIDE (vincristine, ifosfamide, doxorubicin, etoposide) chemotherapy was given for five cycles. This was followed by wide local excision of the tumor after informed consent. Using the extended Henry’s approach, the tumor was exposed and it was found to be extensively involving the flexor carpi radialis and brachioradialis. Histopathology confirmed extraosseus EES involving the flexor muscle of forearm with poor response to chemotherapy (necrosis less than 50%). The patient then received five cycles of VIDE adjuvant chemotherapy and radiotherapy (RT) for five weeks. Thereafter, the patient lost to follow-up and again presented after six months with a fungating mass involving the whole of the right forearm with the involvement of the neurovascular bundle leading to the unresectability of the tumor (Figure 4). After informed consent, an above-elbow amputation was done. The metastatic workup was unremarkable.

At the two-year follow-up, there were no clinical or radiological signs of recurrence or metastasis and the amputation stump was healthy. The patient was explained about the need for continued and regular follow-up.

## Discussion

Angervall and Enzinger first described EES in 1975 [3]. Skeletal/extraskeletal ES, primitive neuroectodermal tumor, and Askin tumors with characteristic karyotype abnormality involving translocation between chromosomes 11 and 22 in common constitute the ES group of tumors. CD99 (a 32-kDa cell surface glycoprotein encoded by the MIC2 gene) positivity is seen in these tumors [4].

Patients with ES generally present in the second decade of life with a rapidly growing mass associated with pain. It is seen more commonly in males. Radiographic findings of EES include increased soft tissue density without any bony involvement. Calcification in the mass is identified only in 25% of the cases. MRI helps to evaluate the extent and internal characteristics of the tumor and its relation with the neurovascular bundle [5]. The areas of hemorrhage within the tumor show different signal intensities depending on the stage of the degradation of blood products. The metastatic workup is vital before proceeding to the definitive management of the tumor [6]. Histopathologically, this group has crowded sheets of small round blue cells or lobules of such cells divided by a small amount of fibrous stroma [7].

Diagnosis in the early stages and prompt treatment are necessary to prevent the regional and distant spread of EES [5]. Neoadjuvant chemotherapy followed by aggressive surgical excision followed with or without RT is the preferred treatment. The goal of surgery should be a three-dimensional tumor-free margin [8]. Although EES is very radiosensitive, surgical advancements and the risks associated with radiation (secondary malignancies) have reduced the reliance upon radiation [9]. Some authors have stated the importance of preoperative RT for successful local treatment in spinal ES [10]. Definitive RT is indicated only when an intralesional resection is possible. Postoperative RT can play a role in cases with a poor histologic response. Postoperative follow-up MRI with contrast administration should be done to detect local recurrence [11]. Regular and long follow-up is necessary to monitor for recurrence and metastasis.

## Conclusions

EES of the forearm is a rare entity that generally presents in the second decade of life. Radiographic and metastatic workup and biopsy are vital before proceeding to the definitive management of the tumor. Early detection and treatment are necessary to prevent recurrence and metastasis.
